# Integration of Proteomic and Lipidomic Analysis Reveals Potential Markers of Insulin Resistance in Young Children With Obesity

**DOI:** 10.1155/pedi/9918136

**Published:** 2025-10-10

**Authors:** Lujie Liu, Jing Zhou, Shuang Guo, Biyao Lian, Hongai Zhang, Yanying Dong, Yuesheng Liu, Shunming Zhang, Chunyan Yin

**Affiliations:** ^1^Department of Pediatrics, The Second Affiliated Hospital of Xi'an Jiaotong University, Xi'an, Shaanxi, China; ^2^Department of Neonatology, Shanghai General Hospital Affiliated to Shanghai Jiao Tong University School of Medicine, Shanghai, China; ^3^Blood Transfusion Department, The Second Affiliated Hospital of Xi'an Jiaotong University, Xi'an, Shaanxi, China; ^4^School of Public Health, Xi'an Jiaotong University Health Science Center, Xi'an, Shaanxi, China

**Keywords:** children obesity, insulin resistance, lipidomic, proteomic

## Abstract

**Objective:**

This study aimed to identify novel proteomic and lipidomic biomarkers of insulin resistance (IR) in young children with obesity and to assess the ability of hub lipids and proteins in the diagnosis of IR.

**Methods:**

The discovery cohort consisted of 50 prepubertal children, including 30 children with obesity and 20 lean. The validation cohort included 25 children with obesity and IR (obese-IR) and 25 children with obesity without IR (obese-NIR). Fasting plasma was collected from all participants for Olink proteomics and untargeted lipidomics. Pearson correlation analysis was used to identify proteins and lipids associated with IR, and area under the receiver operating characteristic (AUROC) was applied to compare the ability of the identified proteins and lipids with traditional indices in the diagnosis of IR.

**Results:**

In the discovery cohort, a total of 15 lipids and 10 proteins had significant correlation with IR. In the validation cohort, protein fatty acid binding protein 4 (FABP4) and gene serpin family E member 1 (PAI) were overexpressed in obese-IR children compared to obese-NIR children, while insulin like growth factor binding protein 1 (IGFBP-1) and paraoxonase 3 (PON3) were lower in the IR group than in the obese-NIR group; five lipids including sphingosine (d16:0), coenzyme (Q8), ceramides phosphate (d42:2), phosphatidylethanolamine (37:2e), and phosphatidylcholine (18:1e_16:0), showed significant (*p* < 0.05) change in obese-IR children compared to obese-NIR children. In addition, the AUC-ROC was 0.89 for IGFBP-1, 0.81 for PON3, and 0.65 for PAI. The ability of IGFBP-1, PON3, and PAI to diagnose IR was better than that of adiponectin and leptin. The AUROC of phosphatidylcholine (18:1e_16:0) and coenzyme (Q8) were 0.80 and 0.73, respectively, which was significantly higher than the AUROC of triglycerides(TGs), total cholesterol (TC), high-density lipoprotein cholesterol (HDL-C), and low-density lipoprotein cholesterol (LDL-C).

**Conclusion:**

Proteomic and lipidomic analysis can allow for the identification of potential new candidate biomarkers for IR. The ability of novel biomarkers to diagnose IR was better than traditional indicators.

**Trial Registration:**

Chinese Clinical Trial Registry: ChiCTR2300072179

## 1. Introduction

Obesity has become a global public health burden [1]. A growing global epidemic, the incidence of childhood obesity continues to rise. Recent data show that there are 340 million overweight and obese children over the age of 5 worldwide. In 2019, the prevalence of childhood obesity was about 115 million children worldwide [2]. Early onset obesity is a clear risk factor for adult obesity, as only a small number of them can lose weight and reach their ideal weight [3]. Persistent weight gain makes them vulnerable to abdominal obesity and increases the risk of comorbidities and reduced life expectancy [4]. Most metabolic comorbidities are evident in obese adults, but are often subtle or even undetectable in most young (preadolescent) obese children. In fact, insulin resistance (IR) is usually the first metabolic abnormality diagnosed in obese children, and is the major risk factor for the development of comorbidities such as impaired glucose tolerance, metabolic dysfunction-associated steatotic liver disease, type 2 diabetes, and polycystic ovary syndrome. Therefore, IR is particularly important in the interpretation of obesity-associated metabolic abnormalities in children [5].

Currently, available biomarkers for predicting IR, especially in young (preadolescent) obese children, are limited. Impaired adipokine synthesis and secretion, which can be detected even in young obese children, contributes to the development of IR [6]. Obesity induces changes in serum levels of insulin-sensitizing adipokines and also creates a generalized proinflammatory environment by increasing circulating levels of resistin, interleukin 6 (IL-6), and tumor necrosis factor-α (TNF-α) [7–9]. Therefore, there is a lack of comprehensive protein markers to predict IR in most young obese children. A similar situation is observed with regard to the obesity-related impairment of lipid metabolism. Lipids are the major components of membrane structures in organisms (such as the outer membrane, mitochondria, endoplasmic reticulum, and exosomes), as well as small signal molecules and energy carriers [10]. The dysfunction of lipid metabolism is closely related to the onset and development of various diseases. In recent years, a few studies have investigated these plasma lipids in relation to obesity or metabolic disorders [11]. According to Rauschert et al. [12] the dysregulation of the balance between sphingolipids (SPs) and glycerophospholipids results in a lipotoxic insult relevant to the pathophysiology of common metabolic diseases such as obesity, diabetes, or IR. Therefore, it may be of particular interest to identify differences between the lipid profiles of obese and normal weight very young obese children and determine whether these profiles are associated with the risk of developing IR.

The aims of this study were: (1) to investigate the influence of obesity and weight loss on the plasma proteomic and lipidomic profile of young obese children. (2) To identify the proteome and lipidome associated with IR in young obese children. (3) To evaluate the differences in proteome and lipidome between young obese children with or without IR.

## 2. Materials and Methods

### 2.1. Ethical Consideration

The study was performed in agreement with the Declaration of Helsinki principles. Ethical approval was obtained from the Second Affiliated Hospital of Xi'an Jiaotong University (Number 2022245). All participants and their parents gave free written informed consent to participate.

### 2.2. Study Population

#### 2.2.1. Discovery Cohort

From May to September 2021, we selected 30 prepubertal (Tanner stage I) obese children (body mass index [BMI] > +2 SDS according to Chinese standards) who visited the pediatric endocrinology department at the Second Affiliated Hospital of Xi'an Jiaotong University [13]. Their mean age at recruitment was 9.88 ± 1.13 years (range 7.53–12.25 years) and their mean BMI was 3.13 ± 0.42 SDS. The 20 prepubertal lean children were recruited from a children's health clinic during the same period when they attended the usual health examination. Their mean age at recruitment was 9.85 ± 1.37 years (range 6.84–11.59) and their mean BMI was 2.61 ± 0.33 SDS. All obese children participated in a 4-week family-based lifestyle intervention; only 22 participants who lost more than 0.5% of their body weight during the 4-week intervention were considered for this study. Fasting blood samples were immediately processed into plasma and stored at −80°C at baseline and the 4-week follow-up.

#### 2.2.2. Validation Cohort

A total of 25 obese children with IR (obese-IR) and 25 children without IR (obese-NIR) attending the pediatric endocrinology department of the Second Affiliated Hospital of Xi'an Jiaotong University were used for further validation. Fasting plasma was collected from all children for further validation of discovery cohort hub proteins using an ELISA kit and for discovery cohort hub lipids using targeted lipidomic analysis.

#### 2.2.3. Family-Based Lifestyle Intervention

All obese children completed a 4-week family-based lifestyle intervention supervised by a multidisciplinary team (pediatric endocrinologist and dietitian). The program featured personalized dietary modification with total daily caloric intake reduced by 350 kcal (~15% baseline reduction), maintaining macronutrient distribution at 50%–65% carbohydrates, 15%–20% fat, and 20%–30% protein. Participants received portion-control tools and digital food diaries for real-time logging. The exercise component prescribed 60 min of daily activity (5 days/week), combining aerobic exercise (40 min at 60%–75% heart rate), resistance training (20 min), and motor-skill development. Compliance was ensured through weekly video consultations reviewing food diaries and activity tracker data (Xiaomi Mi Band 4), supplemented by monthly behavioral workshops addressing parental modeling and screen-time reduction. Standardization was achieved using prerecorded exercise videos and quantifying dietary adherence as <10% deviation from macronutrient targets via DietPro 9.0 software analysis, minimizing interindividual variation throughout the intervention period.

#### 2.2.4. Definition of IR

Homeostasis model assessment of IR (HOMA-IR) index was used to assess the status of IR. HOMA-IR = fasting insulin (mU/L) × fasting glucose (mmol/L)/22.5. Different cut-off values according to pubertal phases were employed to define IR (prepubertal >2.5, pubertal >4.0) [14].

#### 2.2.5. Biochemical Measurements

Plasma lipid profile (triglycerides [TGs], total cholesterol [TC], high density lipoprotein cholesterol [HDL-C], low density lipoprotein cholesterol [LDL-C], and very low density lipoprotein cholesterol [VLDL-C]) and glucose were measured in blood samples using a biochemical automatic analyzer (Hitachi 7060, Tokyo, Japan). Insulin, adiponectin, leptin, IL-6, and TNF-α levels were measured in separate aliquots of each sample using commercial assays.

#### 2.2.6. Lipidomic Analysis

Lipidomic data were obtained using liquid chromatography with electrospray ionization mass spectrometry (LC-ESI-MS). Details of the experimental protocols, including sample preparation and spectroscopy, have been described previously [15]. Analyst 1.6.3 software (SCIEX) was used for data acquisition and processing.

#### 2.2.7. Targeted Lipidomic Analysis

In total, 40 μL of plasma sample and 5 μL of internal standards purchased from Avanti. Hex2Cer(d16 : 1_16 : 0), CerP(t40 : 2), PC(15 : 0_18 : 1), WE(17 : 0), PE(37 : 2e), PC(40 : 4e), PC(18 : 1e_16 : 0), ChE(2 : 0), CerP(d42 : 2), SM(d44 : 5), SPH(t16 : 0), Co(Q8). PC(18 : 1e_16 : 0), SPH(d16 : 0), and PS(27 : 0_11 : 2) were added to 300 μL methanol, 500 μL chloroform, and 250 μL ultrapure water in 4 mL glass tubes. Targeted lipidomic analysis was performed using ultra-high performance liquid chromatography coupled to a triple quadrupole mass spectrometer (UPLC-QqQ-MS/MS, ABI 6500, USA).

#### 2.2.8. Proteomic Analysis

The Olink metabolic and cardiovascular panel, which contains 92 target proteins, was used to analyze samples from 30 obese children, 22 weight-loss children, and 20 lean children. Each target protein was recognized by double antibody labeling and coupled to its complementary DNA barcode, which was then quantified using a high-throughput microfluidic real-time PCR instrument, Biomark HD (Fluidigm, South San Francisco, CA). The final assay readout was provided as normalized protein expression values, which is an arbitrary log2 scale unit corresponding to higher protein levels.

#### 2.2.9. GO Enrichment Analysis and Pathway Enrichment Analysis

The GO and KEGG enrichment analyses were performed using the R software package. The “ggplot2” R tool was used to visualize the findings of GO and KEGG enrichment analysis, and the top 20 GO terms and KEGG pathways were shown as a bubble chart.

#### 2.2.10. Statistical Analysis

Statistical analysis of clinical data was performed using SPSS 23.0 and GraphPad Prism 9.0 software. Data normality was assessed using the Shapiro–Wilk test. Normally distributed clinical data are expressed as mean ± standard error of mean (SEM); categorical data as count (percentage). Group comparisons employed Student's *t*-test (parametric) or Mann–Whitney *U* test (nonparametric) for continuous variables, and chi-squared test for categorical variables. For more than two groups, the one-way ANOVA and the Kruskal–Wallis tests were used to determine differences. In addition, post hoc test (Games–Howell) was performed to compare data from subgroups. Associations between clinical and metabolic variables were examined using Pearson's or Spearman's correlation. For Omics data, differential abundance analysis for Omics data was performed using Wilcoxon rank-sum tests. Resulting *p*-values underwent Benjamini–Hochberg false discovery rate (FDR) correction, with significance defined as FDR < 10%. Multivariate statistical analyses were also performed, including principal component analysis (PCA). Cluster analysis using only significant features was applied to the data using Euclidean distance and complete linkage clustering, presented as a heat map together with the clinical data. A *p*-value < 0.05 was considered statistically significant. All calculations were performed using MetaboAnalyst R in the R environment, and the ggplot2 package was used for graphical visualization in the study.

## 3. Results

### 3.1. Characteristics of Discovery Cohort

A total of 30 obese children and 20 age-matched lean children were included in the study. All obese children received lifestyle interventions, and after 4 weeks, 20 children successfully lost more than 0.5% of their body weight.  Table 1 shows the characteristics of the study participants. There were no significant differences in gender, pubertal stage, or age between the three groups. BMI, SDS-BMI, weight, hip circumference (HC), waist circumference (WC), and waist-hip ratio (WHR) were comparable in the three groups. Levels of systolic blood pressure (SBP), HOMA-IR, and TG were higher, while HDL-C was lower in obese children compared with lean children. In addition, SBP, HOMA-IR, and TG decreased significantly after the intervention.

### 3.2. Lipidomic Alterations Associated With IR in Obese Children

**Figure 1:**
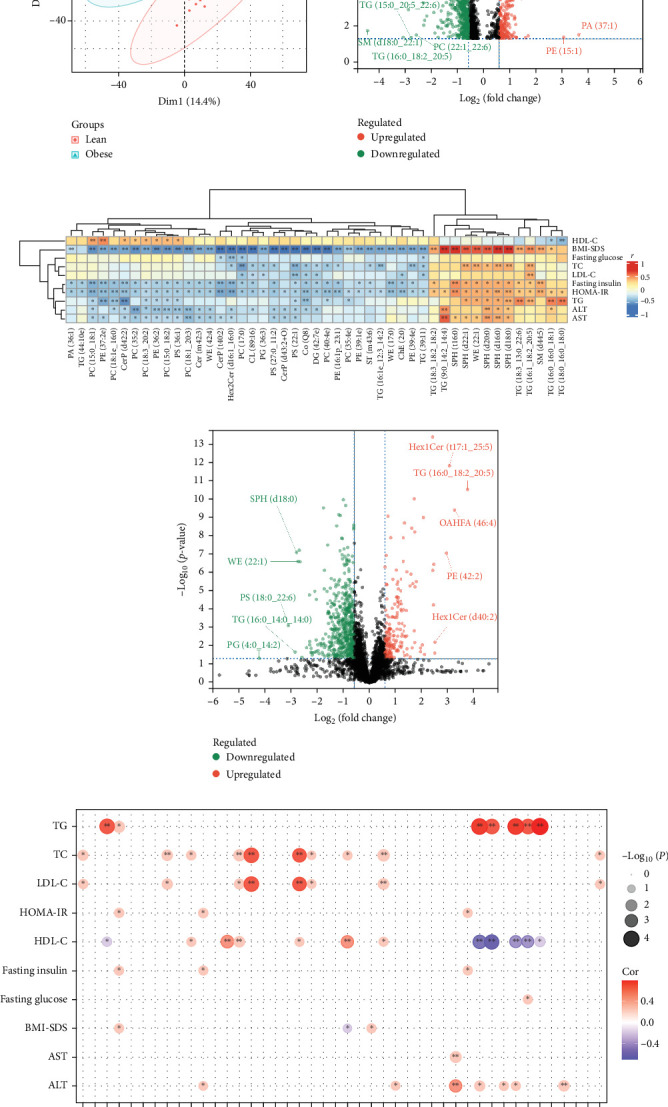
Lipidomic alterations associated with IR in obese children. (A) Supervised PCA model shows discrimination of lipidomics between obese and lean children. (B) Volcanic visualization of lipidomic between obese and lean children. (C) Correlation between clinical indicators and lipidomic in obese children. (D) Volcanic visualization of lipidomic between the pre- and postintervention groups. (E) Changes in lipidomic associated with insulin resistance in obese children before and after weight loss. *⁣*^*∗*^*p* < 0.05, *⁣*^*∗∗*^*p* < 0.01.

To detect the significantly altered lipid metabolites in obese children compared to lean children. PCA analysis was performed with two predictor components. The PCA plots could be completely separated into two clusters, illustrating that the plasma lipid profiles were significantly altered in obese children (Figure 1A). In the obese children, the mean abundance of 181 lipids showed a >twofold increase, and 62 lipids showed a >twofold decrease compared to lean children. Figure 1B shows the volcano plot of differential lipid species and indicates the top 10 lipids with remarkable change in log_2_FC and *p* < 0.05. The lipids (VIP > 1, *p* < 0.05) between the obese and lean groups were also analyzed and a total of 138 lipids were identified. Correlation analysis was performed between these lipids and clinical indicators. Among them, we found that 15 lipids with significant correlation with HOMA-IR, which were Hex2Cer(d16:1_16:0), CerP(t40:2), PC(15:0_18:1), WE(17:0), PE (37:2e), PC(40:4e), PC(18:1e_16:0), PE(16;1p_23:1) ChE(2:0), CerP(d42:2), SM(d44:5), SPH(t16:0), SPH (d18:0), Co(Q8), and SPH(d16:0) (Figure 1C).

### 3.3. Effects of Weight Loss on IR-Associated Lipidomic Profile in Obese Children

We then performed a univariate analysis with all the lipids found before and after weight loss in obese children. A volcano plot (Figure 1D) shows the most significant differences in lipids, the positive or negative fold change seen in obese children after weight loss. It was noteworthy that weight loss in obese children ameliorated obesity-induced IR and that the correlation between 11 lipids namely Hex2Cer(d16 : 1_16 : 0), CerP(t40 : 2), WE(17 : 0), PE(37 : 2e), PC(40 : 4e), PC(18 : 1e_16 : 0), CerP(d42 : 2), SM(d44 : 5), Co(Q8), SPH(d16 : 0), SPH(d18 : 0), and IR. Weight loss also increased the levels of ChE(2 : 0) and PC(15 : 0_18 : 1), and the correlation between these two lipids and the HOMA-IR changed from negative to positive. SPH(t16:0) levels decreased after weight loss and the correlation with the IR index became weaker (Figure 1E).

### 3.4. Protein Alterations Associated With IR in Obese Children

**Figure 2:**
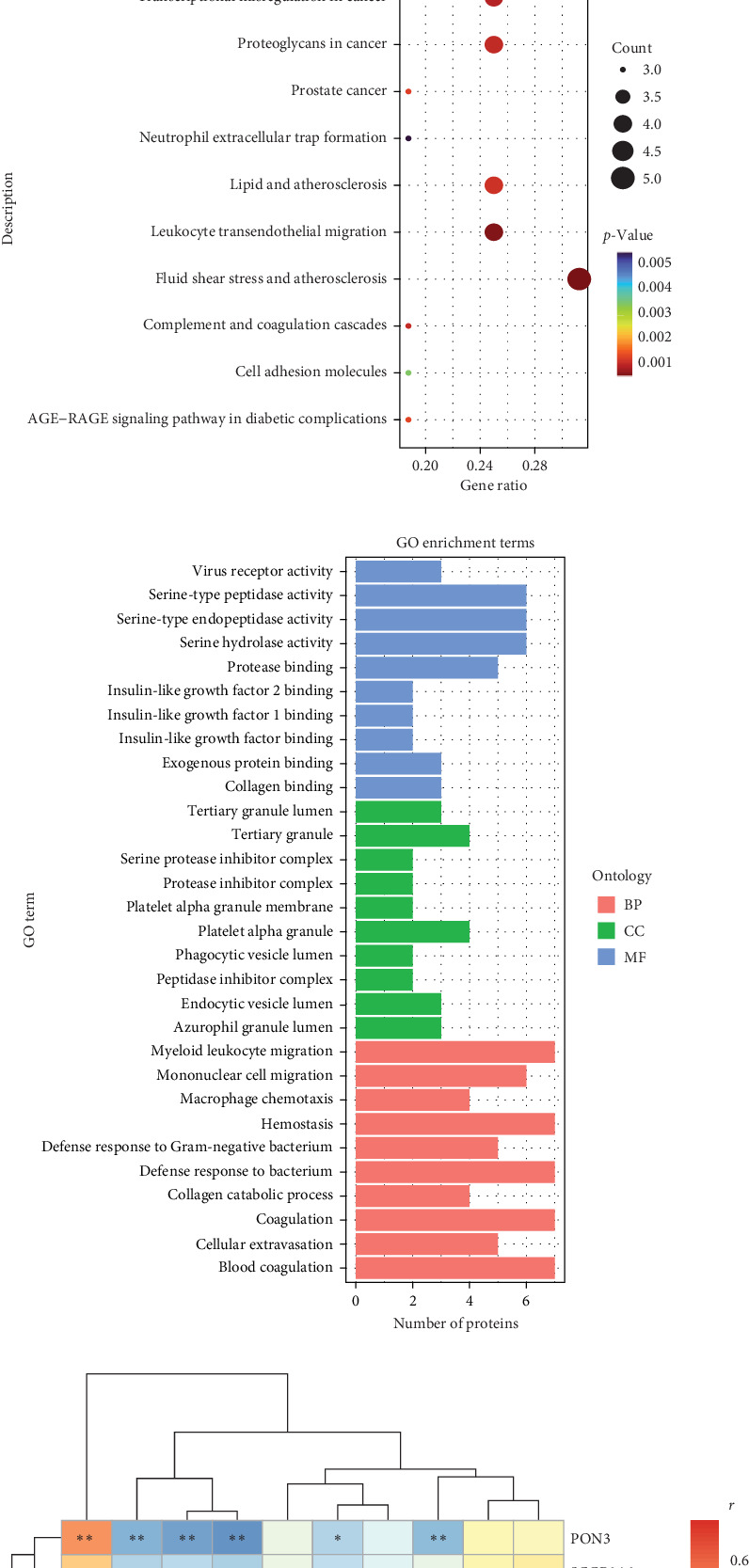
Proteins alterations associated with IR in obese children. (A) Supervised PCA model shows discrimination between the obese and lean children. (B) Ring heatmap of differentially expressed proteins in obese children. (C) KEGG enrichment analysis based on the background of 92 metabolic and cardiovascular related proteins. (D) GO enrichment analysis based on the background of 92 metabolic and cardiovascular related proteins. (E) Correlation between clinical indicators and proteins in obese children.

The Olink metabolic and cardiovascular panel was used to detect differences in protein expression between obese and lean samples. PCA analysis was performed with two predictor components. The PCA plots could be completely separated into two clusters, illustrating that the plasma protein profiles were significantly altered in obese children (Figure 2A). A total of 31 differentially expressed proteins (DEPs) were identified between obese and lean children, of which 23 proteins were upregulated and eight proteins were downregulated in the obese children. A ring heatmap of these DEPs is shown in Figure 2B. GO and KEGG enrichment analysis was used to further investigate the potential functions of the DEPs. Proteins were mainly enriched in GO terms such as blood coagulation, myeloid leukocyte migration, coagulation, hemostasis, and defense response to bacteria (Figure 2C). KEGG pathways such as fluid shear stress and atherosclerosis, leukocyte transendothelial migration, transcriptional misregulation in cancer, complement and coagulation cascades, and lipid and atherosclerosis were significantly enriched (Figure 2D). Next, the association between plasma DEPs and clinical features, in particular the correlation with IR, was analyzed (Figure 2E). Four proteins, fatty acid binding protein 4 (FABP4), CH3IL1, PAI, and RARRES2, were positively associated with IR. Six candidate markers, paraoxonase 3 (PON3), SCGB3A2, KLK6, insulin like growth factor binding protein 1 (IGFBP-1), IGFBP-2, and NT.proBNP were negatively associated with IR.

**Figure 3:**
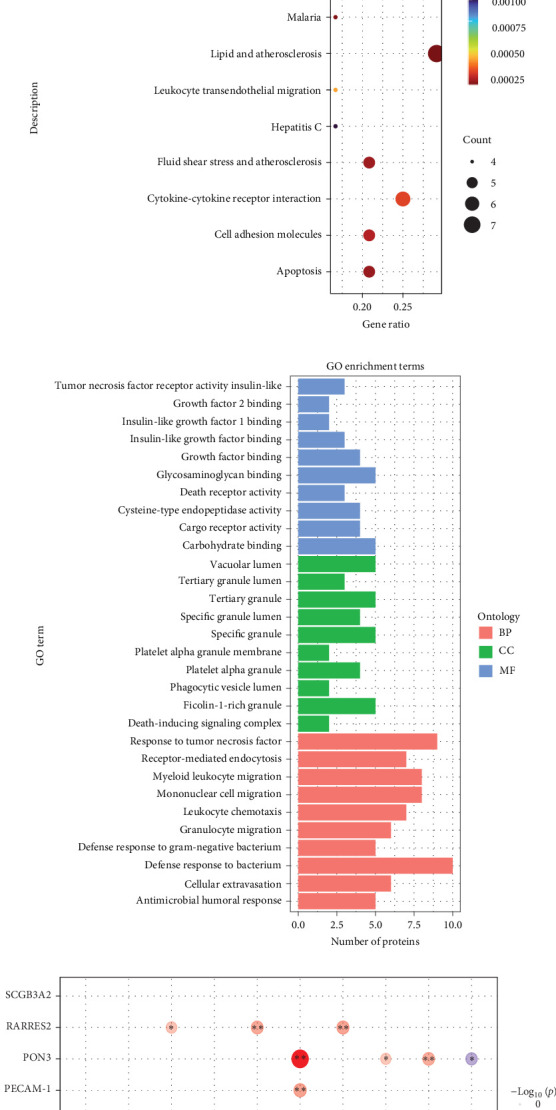
Effects of weight loss on IR-associated proteins in obese children. (A) Volcanic visualization of metabolic and cardiovascular related proteins between the pre- and postintervention groups by Olink analysis. (B) KEGG enrichment analysis based on the background of metabolic and cardiovascular related proteins before and after weight loss in obese children. (C) GO enrichment analysis based on the background of metabolic and cardiovascular related proteins before and after weight loss in obese children. (D) Changes in proteins associated with insulin resistance in obese children before and after weight loss. *⁣*^*∗*^*p* < 0.05, *⁣*^*∗∗*^*p* < 0.01.

We compared the expression levels of proteins between the pre- and postintervention groups by Olink analysis. We identified 39 proteins that were differentially expressed between the pre- and postintervention groups, including 37 upregulated proteins and two downregulated proteins (Figure 3A). We also performed GO and KEGG enrichment analyses to investigate the potential functions of the proteins after weight loss. In the GO enrichment analysis, the results showed that death receptor activity, tumor necrosis factor receptor activity, tumor necrosis factor response, and mononuclear cell migration were enriched (Figure 3B). Furthermore, KEGG enrichment analysis suggested that lipid and atherosclerosis, cytokine–cytokine receptor interaction, and TNF signaling pathway were enriched (Figure 3C). In addition, we focused on the effects of weight loss on 10 DEPs in obese children that are closely associated with IR. After weight loss, the association between HOMA-IR and other proteins disappeared except for IGFBP1, CHI3L1, and RARRES2. The correlation of IGFBP1, CHI3L1, and RARRES2 with the HOMA-IR became weaker (Figure 3D).

### 3.5. Clinical Characteristics of the Validation Cohort

Validation cohort including 25 obese-IR children and 25 obese-NIR children. Demographic and general clinical information is summarized (Table 2). There were no statistical differences in gender, pubertal stage, or age between the two groups. Children with IR had higher weight, HC, WC, and WHR than obese-NIR, and HOMA-IR, TG, TC, HDL-C, and adiponectin levels were elevated in obese-IR than in obese-NIR.

### 3.6. Validation of Top Differentially Expressed Lipids and Proteins in Validation Cohort

**Figure 4:**
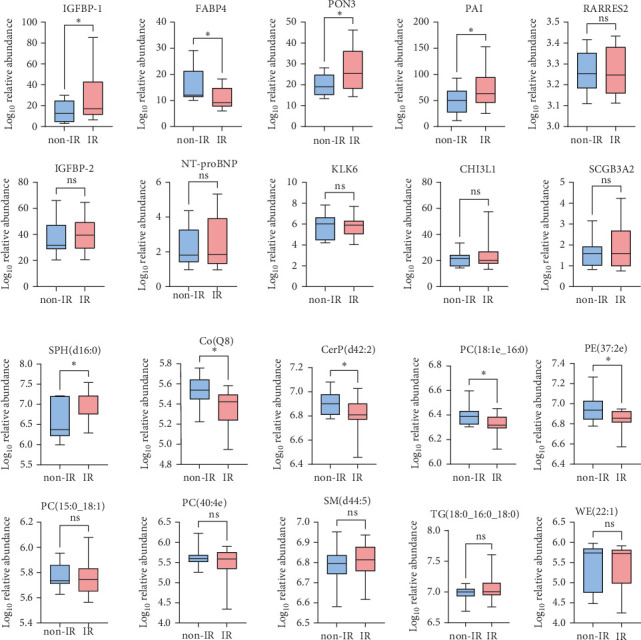
Validation of IR-associated lipids and proteins in obese children with IR. (A) Validation of lipids significantly altered in obese children and associated with HOMA-IR in obese children with IR. (B) Validation of proteins significantly altered in obese children and associated with HOMA-IR in obese children with IR, *⁣*^*∗*^*p* < 0.05. ns, not statistically significant.

The comparison of the serum proteomic profile between obese-IR and obese-NIR allowed the analysis of 10 proteins significantly altered in obese children and associated with IR by ELISA. Among these, FABP4 and PAI were overexpressed in IR compared to non-IR obese children, IGFBP-1 and PON3 were significantly lower in the IR group than in the non-IR group, whereas the expression of the remaining six proteins was unchanged in IR (Figure 4B).

We also identified the lipids significantly altered in obese children and associated with IR in the validation cohort by targeted analysis of lipids. Among the top 15 lipids, five compounds; including SPH(d16:0), Co(Q8), CerP(d42:2), PE(37:2e), and PC(18:1e_16:0), showed significant (*p* < 0.05) change in obese-IR compared to obese-NIR (Figure 4A).

### 3.7. Logistic Regression Analysis to Identify Hub Lipids and Proteins Associated With IR

The forward stepwise model was used in the logistic regression analysis. We chose HOMA-IR values as the dependent variable and SPH(d16:0), Co(Q8), CerP(d42:2), PE(37:2e), PC(18:1e_16:0), TG, TC, HDL-C, and LDL-C as the independent variables. We found that PC(18:1e_16:0), PE(37:2e), and Co(Q8) were the protective factors against IR after adjustment for age, sex, SDS-BMI, and WC (Table 3).

We performed a logistic regression analysis with IGFBP-1, FABP4, PON3, PAI, adiponectin, and leptin as independent variables. This showed that only IGFBP-1, PON3, PAI, and adiponectin were significantly associated with IR after adjustment for age, sex, SDS-BMI, and WC (Table 4).

### 3.8. The Ability of Hub Lipids and Proteins in the Diagnosis of IR

To compare the ability of lipid metabolites with traditional dyslipidemia indices in the diagnosis of IR. Receiver operating characteristic (ROC) analysis of PC(18:1e_16:0), PE(37:2e), Co(Q8), and TG, HDL-C and LDL-C was performed. The area under ROC (AUROC) of individual lipids, especially PC(18:1e_16:0) and Co(Q8) were 0.80 and 0.73 respectively, which was significantly higher than the AUC-ROC of TG, HDL-C and LDL-C.

**Figure 5:**
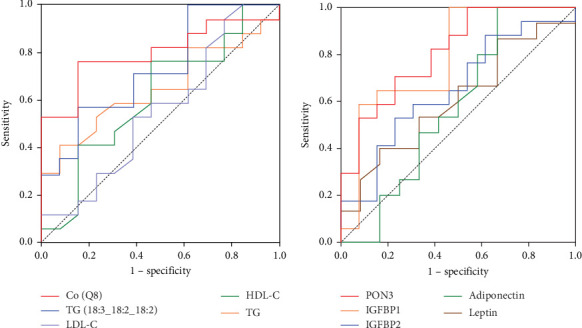
Ability of hub lipids and proteins to diagnose IR. (A) The ROC curves of individual and AUC of hub lipids in obese children with IR. (B) The ROC curves of individual and AUC of DEPs in obese children with IR.

The Figure 5 presents the AUC-ROC of IGFBP-1, PON3, PAI, and the IGFBP-1 level was found to be a statistically significant marker in discriminating the obese-IR from the obese-NIR at a rate of 63.2%. The PON3 level discriminated the obese-IR from the obese-NIR at a rate of 52.4%. The PAI value discriminated the obese-IR from the obese-NIR at a rate of 36.1%. The AUC-ROC for IGFBP-1 was 0.89, for PON3 0.81, and for PAI 0.65. The ability of IGFBP-1, PON3, and PAI to diagnose IR was better than that of adiponectin and leptin.

## 4. Discussion

IR is a key step in the progression from obesity to type 2 diabetes. The cause of IR is not limited to impaired insulin signaling but also involves the complex interplay of multiple metabolic pathways [15]. Several previous studies reporting altered lipids and proteins have shed new light on the role of metabolites in IR. However, not all obese with IR have traditional abnormal clinical plasma indicators. Given the contradictory relationship, novel biomarkers for obesity-associated IR are warranted.

In the present study, we observed that 14 lipids were significantly associated with HOMA-IR in obese children (*r*>0.4, *p* < 0.05), and they were Hex2Cer(d16:1_16:0), CerP(t40:2), CerP(d42:2), PC(15:0_18:1), PC(40:4e), PC(18:1e_16:0), WE(17:0), PE(37:2e), ChE(2:0), SM(d44:5), SPH(t16:0), SPH(d16:0), Co(Q8), and PS(27:0_11:2), and after weight loss, the above lipids changed significantly as IR improved. Specifically, the correlation between 11 lipids Hex2Cer(d16 : 1_16 : 0), CerP(t40 : 2), WE(17 : 0), PE(37 : 2e), PC(40 : 4e), PC(18 : 1e_16 : 0), CerP(d42 : 2), SM(d44 : 5), Co(Q8), SPH(d16 : 0), and PS(27 : 0_11 : 2) and HOMA-IR disappeared, but weight loss increased the levels of ChE(2:0) and PC(15:0_18:1). In addition, the correlation between these two lipids and the IR index changed from negative to positive. SPH(t16:0) levels decreased after weight loss and the correlation with IR became weaker. The above results suggest that these lipids may play an important role in obesity-related IR. Regarding glycerophospholipids, we found that PC(15:0_18:1), as an important molecular species of PCs, increased after the intervention. Our results are in agreement with those of Chang et al. [16] which showed that PC(40:6) and PC(40:4) were decreased in obesity, and LPC(20:2) was increased after intervention. The fact that SPH(t16:0) decreased after the intervention could mean that weight interventions have a positive effect on obese children. SP metabolism is altered in obese individuals because there is an increase in free fatty acids (FFAs), which leads to an increase in palmitoyl-CoA after an increase in ceramide production via de novo pathway [17].

Furthermore, our results in the validation cohort show that among 14 lipids, SPH(d16:0), Co(Q8), CerP(d42:2), PE(37:2e), and PC(18:1e_16:0) were significantly altered in obese children with IR. SPs, including ceramides and sphingomyelins, may indicate a possible metabolic disorder and IR [18]. A study by Hannun and Obeid [17] showed that ceramide derived from saturated fat may be a major contributor to IR. Elevated PC levels have been suggested to increase coronary heart disease and mortality. Previous reports have observed that PC is positively associated with IR, type 2 diabetes, and cardiovascular disease in obesity [19, 20]. CoQ is a mitochondrial cofactor and antioxidant that is synthesized and localized in the inner mitochondrial membrane (IMM). Diaz-Vegas et al. [21] reported that a strong inverse relationship between mitochondrial CoQ and ceramide levels is closely linked to the control of cellular insulin sensitivity.

On the other hand, plasma inflammation-related protein profiles were comprehensively identified between obese and lean children by Olink proteomics, and 10 significantly altered DEPs were identified. The DEPs were enriched in inflammation and immune response and were significantly related to HOMA-IR. After weight intervention, the correlation between IGFBP1, CHI3L1, and RARRES2 and HOMA-IR was attenuated, and the correlation between other proteins and HOMA-IR disappeared. Our results suggest that the above proteins may contribute to the development of IR. Furthermore, in the validation cohort, we found that four proteins (IGFBP-1, FABP4, PON3, and PAI) of the 10 DEPs were elevated in IR children. The results of our study are consistent with other similar studies. FABP4 is a cytoplasmic fatty acid chaperone that has been implicated in the development of IR [22], and deletion of the FABP4 gene protected mice from diet-induced IR [23]. Reinehr et al. [24] and others have reported significant positive correlations of IGFBP-1 with several indices of insulin sensitivity in obese children, and IGFBP-1 may be an independent risk factor for IR and increased risk of type 2 diabetes in children, even at a young age [ 25]. The remaining two DEPs (PON3 and PAI) were novel findings in this study. The observed downregulation of PON3 in obese-IR children aligns with its known role in mitigating oxidative stress and inflammation key drivers of IR. As demonstrated by Rull et al. [26], serum PON3 concentration is positively associated with insulin sensitivity. Mechanistically, PON3 attenuates lipid peroxidation in mitochondrial membranes, preserving insulin signaling fidelity. Its deficiency permits accumulation of oxidized LDL, which activates pro-inflammatory pathways and serine kinases that impair insulin receptor function [27]. This establishes a direct link between PON3 depletion, oxidative damage, and IR pathogenesis. Elevated PAI-1, consistently observed in IR states, promotes IR by inhibiting fibrinolysis which induces microvascular thrombosis and impairs capillary perfusion in insulin-sensitive tissues, reducing glucose uptake [28].

We also compared the diagnostic efficacy and advantages of lipids with traditional clinical plasma lipid parameters. The results show that PC(18:1e_16:0), PE(37:2e), and Co(Q8) were the protective factors for IR. PC(18:1e_16:0) and Co(Q8) had higher diagnostic values than TG, HDL-C, and LDL-C. Similarly, we found that the ability of IGFBP-1, PON3, and PAI to diagnose IR was better than adiponectin and leptin. PC(18:1e_16:0), Co(Q8) IGFBP-1, PON3, and PAI may serve as potential diagnostic plasma biomarkers for obesity-related IR. Based on the reported literature, inflammatory protein and lipid metabolites may induce the inflammatory or immune response through a very complex mechanism, thereby contributing to the development of IR [29, 30].

To our knowledge, the present study is the first to combine untargeted lipidomics and Olink proteomics to assess lipid and protein profiles in normal weight children, obese children, and obese children after weight loss. We also identified novel proteomic and lipidomic biomarkers of IR in young obese children and found that the ability of novel biomarkers to diagnose IR was better than traditional indicators. Future monitoring of key protein biomarkers via low-cost ELISA assays promises to improve IR diagnosis in children.

There are some limitations to this study. First, the sample size was relatively small, making it difficult to detect small effects. The main reason for this is that it was not possible to collect a large enough sample due to refusal by the child and parents and various technical reasons during the blood collection. Second, while our findings reveal significant associations between specific proteins and lipids with IR in this pediatric cohort, the causative mechanisms underlying these relationships remain to be elucidated. Future mechanistic studies using in vitro or animal models are warranted to determine whether these biomarkers play direct functional roles in IR pathogenesis or represent secondary consequences of metabolic dysregulation. Third, this was a single-center study. The results of our study need to be replicated in other centers so that the biomarkers for IR diagnosis can be widely disseminated. However, our results were validated in the validation cohort. Furthermore, our findings cannot be extrapolated to other populations.

In conclusion, using the untargeted lipidomics and Olink proteomics approach, a paradigm of protein and lipid metabolic biosignatures was identified that could discriminate IR from obese children. Our study provides novel biomarkers for the prediction of obesity-related IR.

## Figures and Tables

**Table 1:** The characteristics of discovery cohort.

Variables	Lean (20)	Obese (30)	Postintervention (22)	*p*
Age (year)	9.85 ± 1.70	9.88 ± 1.13	9.93 ± 1.54	0.949
BMI (kg/m^2^)	15.08 ± 1.58	27.59 ± 4.36^a^	24.58 ± 4.32^a,b^	~ 0.001
SDS-BMI	2.61 ± 0.33	3.13 ± 0.42^a^	2.97 ± 0.67^a,b^	0.001
Weight (kg)	31.08 ± 4.87	63.67 ± 12.55^a^	57.42 ± 12.25^a,b^	~ 0.001
HC (cm)	75.22 ± 2.60	96.49 ± 6.75^a^	90.57 ± 5.68^a,b^	~ 0.001
WC (cm)	58.51 ± 3.93	84.76 ± 11.93^a^	79.48 ± 9.73^a,b^	~ 0.001
WHR	0.81 ± 0.04	0.97 ± 0.07^a^	0.89 ± 0.05^a,b^	~ 0.001
SBP (mmHg)	96.2 ± 3.9	107.4 ± 10.2^a^	101.7 ± 8.69^a,b^	~ 0.001
DBP (mmHg)	76.4 ± 3.5	78.2 ± 4.1	77.8 ± 5.3	0.576
FPG (mmol/L)	4.47 ± 0.57	5.12 ± 0.45	5.07 ± 0.63	0.648
HOMA-IR	2.64 (1.82, 4.47)	3.63 (2.13, 5.79)^a^	2.78 (1.98, 4.84)^a,b^	~ 0.001

Abbreviations: BMI, body mass index; DBP, diastolic blood pressure; FPG, fasting plasma glucose; HC, hip circumference; HOMA-IR, homeostatic model assessment of insulin resistance; SBP, systolic blood pressure; SDS- BMI, BMI standard deviation score; WC, waist circumference; WHR, waist-hip ratio.

^a^ *p* < 0.05, compared with lean.

^b^ *p* < 0.05, compared with the obesity group.

**Table 2:** Clinical characteristics of the validation cohort.

Variables	Obese-IR (25)	Obese-NIR (25)	*p*
Age (year)	10.45 ± 2.43	10.16 ± 1.78	0.949
BMI (kg/m^2^)	28.32 ± 5.45	24.58 ± 3.27	~ 0.001
SDS-BMI	3.54 ± 045	2.78 ± 0.31	0.002
Weight (kg)	67.45 ± 10.38	58.45 ± 8.32	~ 0.001
HC (cm)	95.37 ± 6.32	86.38 ± 5.49	~ 0.001
WC (cm)	88.471 ± 12.45	80.65 ± 10.73	~ 0.001
WHR	1.02 ± 0.08	0.85 ± 0.07	~ 0.001
FPG (mmol/L)	5.47 ± 0.65	5.08 ± 0.55	0.648
HOMA-IR	3.64(2.67, 4.84)	2.05(1.05, 2.42)	~ 0.001
TC (mmol/L)	4.53 ± 0.84	4.05 ± 0.65	0.037
TG (mmol/L)	1.17 ± 0.42	0.91 ± 0.29	0.048
HDL-C (mmol/L)	2.16 ± 0.56	1.55 ± 0.67	0.036
LDL-C (mmol/L)	2.30 ± 0.37	2.84 ± 0.76	0.280
Adiponectin (ug/mL)	1.76 ± 0.78	2.03 ± 0.96	0.001
Leptin (ng/mL)	49.32 ± 13.08	40.21 ± 11.34	0.089

**Table 3:** Logistic regression analysis using IR as the dependent variable to determine the association with lipids.

Dependent	Independent	Parameter	OR	Wald	*P*
IR (binary: IR vs. non-IR)	SPH(d16:0), Co(Q8), CerP(d42:2), PE(37:2e), PC(18:1e_16:0), TG, TC, HDL-C, and LDL-C	PC(18:1e_16:0)	0.382	12.986	~ 0.001
		PE(37:2e)	0.847	5.449	0.020
		Co(Q8)	0.455	10.938	0.001
		TG	1.783	4.325	0.027

*Note:* Model adjustment for age, sex, SDS-BMI, and waist circumference.

**Table 4:** Logistic regression analysis using IR as the dependent variable to determine the association with proteins.

Dependent	Independent	Parameter	OR	Wald	*P*
IR (binary: IR vs. non-IR)	IGFBP-1, FABP4, PON3, PAI, adiponectin, and leptin	IGFBP-1	0.314	5.07	0.024
		PON3	0.484	4.36	0.037
		PAI	1.484	4.78	0.032
		Adiponectin	1.455	3.53	0.041

*Note:* Model adjustment for age, sex, SDS-BMI, and waist circumference.

## Data Availability

Data are available upon reasonable request from the corresponding author.
